# Predictors of professional help-seeking for emotional problems in Afghan and Iraqi refugees in Australia: findings from the Building a New Life in Australia Database

**DOI:** 10.1186/s12889-019-7673-5

**Published:** 2019-11-08

**Authors:** Shameran Slewa-Younan, Pilar Rioseco, Maria Gabriela Uribe Guajardo, Jonathan Mond

**Affiliations:** 1 Mental Health, Translational Health Research Institute, School of Medicine and Humanitarian and Development Research Initiative, Western Sydney University, Sydney, Australia; 2 Centre for Mental Health, Melbourne School of Population and Global Health, University of Melbourne, Melbourne, Australia; 3 Research Officer, Mental Health, School of Medicine, Western Sydney University, Sydney, Australia; 4 Australian Institute of Family Studies, Melbourne, Australia; 5 Centre for Rural Health, College of Health and Medicine, University of Tasmania, Launceston, Australia; 6 School of Medicine, Western Sydney University, Sydney, Australia

**Keywords:** Refugees, Mental health, Help-seeking, Mental health promotion

## Abstract

**Background:**

Refugees are particularly vulnerable to poor mental health outcomes due to exposure to pre migration trauma and post migration stressors. Research has demonstrated evidence to suggest that the professional help-seeking among refugee groups is low or problematic. This study seeks to examine help-seeking for emotional problems in two large samples of Iraqi and Afghan refugees in Australia.

**Methods:**

This study uses data from two waves of the Building a New Life in Australia, the longitudinal study of Humanitarian migrants. The data was collected face-to-face between 2013 and 2016, among humanitarian migrants. All participants held a permanent protection visa and had arrived in Australia or been granted their visa between period of May to December 2013. The study sample included 1288 participants born in Iraq and Afghanistan (aged 15 and over). In the Wave 3 interview (2015–2016) participants reported on professional help received to deal with emotional problems.

**Results:**

Approximately 36 and 37% of the Iraqi and Afghan groups respectively, reported seeking help for emotional problems. Within the Iraqi group, associations between mental health status, namely general psychological distress and PTSD and help-seeking were found but this was not present in the Afghan group, where age seemed to play a role in help-seeking. Frequency of help received was low with approximately 47% of the Iraqi and 57% of the Afghan groups reporting having received help 5 times or less in the last 12 months.

**Conclusions:**

Findings from this study provide clear directions on areas where culturally tailored mental health promotion programs should target in these two refugee communities. Further, the differences in help-seeking behaviour of these communities should be noted by both clinicians and policy makers as efforts to provide culturally responsive mental health services.

## Background

An unprecedented 68.5 million people around the world have been forcibly displaced [1] and among them are nearly 25.4 million refugees [1] according to the United Nations High Commissioner for Refugees (UNHCR) most recent report [1]. A refugee is a ‘*person who has left their home country as a result of a well*-*founded fear of persecution for reasons of race, religion, nationality, membership of a particular social group or political opinion’* (p. 16) [2]. Refugee resettlement in Australia has increased significantly over the last three years, from a base of 13,750 to the current level of 18, 750 annually [3]. An additional 12,000 refugees from Syria and Iraq were resettled over 2016 and 2017, and these groups in addition to those from Afghanistan continue to make up the bulk of the current intake. This increased intake numbers demonstrate the commitment the Australian government is providing as a response to the global humanitarian crisis.

In Australia, resettled refugees (those granted a permanent protection visa) are provided support services that focus on fostering social and economic participation, personal well-being, independence and community connectedness. Some examples include offering opportunities for education through targeted English tuition and case work coordination. Additionally, they are provided a full range of Australian Government employment services assistance, and also income support, from the date of their arrival in Australia. Another important area of focus in the resettlement of refugees is the provision of appropriate mental health services post resettlement. This is because mental health problems such as posttraumatic stress disorder (PTSD) and major depressive episode (MDE) in particular, are over-represented among refugee populations [4]. For example, in one of the largest meta-analyses undertaken on the mental health of resettled refugees, weighted prevalence rates across the studies were 30.6 and 30.8% for PTSD and depression, respectively [5]. Further, in a study of Iraqi refugees resettled in Western countries, it was found that rates of PTSD varied from 8 to 37.2%, and those of depression from 28.3 to 75% [6]. These rates far exceed the 12-month prevalence rates for PTSD of 6.4% and depression of 4.1% in the general Australian population [7]. In addition to higher rates of mental health problems, research has indicated that rates of professional help-seeking among refugee groups are well below that of individuals with mental health problems in the non-refugee host population [8]. Professional help-seeking has been defined as assistance from professionals who have a legitimate and recognised professional role in providing relevant advice, support and/or treatment [9]. In research conducted by Correa-Velez and colleagues [8, 10], in which the annual, age-standardised hospital admission rates in Victoria for people born in refugee-source countries were compared with those of Australian-born people, participants from a refugee background were 30% less likely to have mental or behavioural admissions than those born in Australia.

Since 2015, our group has sought to explore help-seeking for mental health problems amongst resettled refugee groups in Australia. In an initial investigation, it was noted that only a third of Iraqi refugees with clinically significant PTSD symptom levels reported seeking professional help [11]. Further, when factors associated with help-seeking within the symptomatic subgroup were examined, psychological distress levels were associated with perceived need for help and subsequent help-seeking [12]. Similarly, in a sample of resettled Afghan refugees, it was noted that for every one unit increase in functional disability score it was associated with an 8.8% increase in the odds of seeking help [13]. These findings are not surprising. Previous research, relating to broad range of mental health problems in various study populations, has found that distress and disability are strongly predictive of perceived need for treatment and, in turn, help-seeking, among individuals with these problems [14, 15]. On the other hand, help-seeking and the factors associated with this might be expected to differ for resettled refugees and there is relatively little research addressing these issues among resettled refugees recruited from the general population. Hence the current study is important in providing this information. Nonetheless, these previous community based refugee studies are important as it is helpful to confirm that resettled refugees behave like other populations in this regard, for example knowing that individuals who are not currently experiencing significant impairment or high levels of distress may be unlikely to seek treatment despite being at high risk of this in future. Other studies of non-refugee samples have noted that specific socio-demographic factors such as age and gender in addition to clinical symptomology are also associated with help-seeking in clinical samples of those with depression and anxiety [16, 17], however such associations have not been reported in refugee populations.

A major challenge of these previous studies that have explored help-seeking in refugee populations to date, has been methodological issues associated with, small, non-random and convenience sampling, which has implications for the generalisability of findings [18]. In an effort to develop a more robust understanding of factors influencing professional help-seeking in two key refugee groups being resettled in Australia, those from Afghanistan and Iraq, this study proposes to utilise the data from the Building a New Life in Australia (BNLA) study [19]. The BNLA project is the first and largest longitudinal prospective cohort study of refugees and their families in Australia, and one of the largest in the world [20]. Elucidating factors that are associated with professional help-seeking will allow the development of clearer protocols around mental health promotion programs and provide information to policy makers through to clinicians within this field. With these considerations in mind, the aim of this study was to examine the associative role that socio-demographic variables, trauma exposure and mental health symptomology had on professional help-seeking behaviour in a large sample of Afghan and Iraqi refugees resettled in Australia.

## Method

### Participants

A total of 1288 participants were included in this study, consisting of 803 and 485 resettled refugee participants born in Iraq and Afghanistan, respectively. As part of the inclusion criteria all participants held a permanent protection visa and had arrived in Australia or been granted their visa between period of May to December 2013. Participants were recruited from eleven sites across Australia, encompassing major cities and regional centres. The eleven study sites were selected based on the concentration of humanitarian migrants at the time and accounted for 92% of settlements in this time period. The dataset utilised in this study was extracted from larger project database “Building a New Life in Australia” (BNLA) which included persons from a diverse range of backgrounds and migration pathways [19]. The BNLA sample was randomly selected form the Australian government Settlement Database, which contains information on all humanitarian arrivals to Australia. The BNLA is a longitudinal study that seeks to increase the knowledge base on the factors associated with successful settlement of humanitarian migrants during their first five years in Australia [19]. The BNLA was funded by the Australian Government Department of Social Services (DSS) and managed by the Australian Institute of Family Studies (AIFS). More details of the full cohort have been described elsewhere [19].

### Data collection procedure

All study participants provided voluntary consent to take part in the study.

Recruitment for the BNLA study was based on the Principal Applicants (PA) on the humanitarian visa application. Once the PA had agreed to participate, other family members on the visa application who were aged 15 or over were invited to take part in the study (secondary applicants). Data collection for the full BNLA cohort occurred across five annual Waves and entailed both face-to-face and telephone interview formats. For the purpose of the current study, data collected at wave 1 and wave 3 were utilised determined by either when it was available (e.g. information on help-seeking for mental health problems and traumatic events individually experienced or witnessed were only available in Wave 3) or because it was the closest time point to the time of arrival (e.g. data collected at Wave 1). Wave 1 was collected from October 2013 to March 2014. Wave 3 was collected from October 2015 to February 2016.

Questionnaires were translated into a large number of languages enabling most participants to complete the survey in their native language (in the case of our participants being Arabic, Persian or Dari). The BNLA database contains multiple measures of settlement related issues including sociodemographic characteristics, pre-migration stressors, mental health measures, migration pathways, housing, language, employment, education and related social and economic issues [21]. Variables names as assigned in the data dictionary were utilised in this study [22]. The first and third Waves of the BNLA dataset are publicly available and accessible by authorised researchers who have obtained permission from the DSS.

### Measures

#### Independent variables

##### Socio-demographic characteristics

Respondent’s age, gender, location and pre-migration education attainment were collected at Wave 1. Specifically, education levels were categorised into ‘never attended school’, ‘9 or less years of schooling’, ‘10 or more years of schooling’ and ‘post-school qualification’. Participants’ location at Wave 1 was coded into ‘major cities in Australia’ or ‘regional Australia’ and length of residence in Australia at the time of the first interview (Wave 1) was coded ‘less than 6 months’ or ‘6 months or more’.

Country of birth was used as a proxy for national background. The subsample used in this study included only respondents from Iraq and Afghanistan as they comprised the two largest groups in the dataset. A measure of the importance of religion was included in this study. Respondents who reported having a religion were asked “*How important is religion to you?”* Response options were ‘very important’, ‘quite important’, ‘not very important’, and ‘not important’. For the purpose of the current analysis, response options ‘not very important’ and ‘not important’ were grouped, as well as no religion (only 1 case in the subsample included in this analysis). These data was collected in Wave 1.

A single measure of ‘self-sufficiency’ collected at Wave 1 was included in the present analysis. Respondents were asked how often they had difficulty travelling to places they needed to go. Response options included ‘always’, ‘most of the time’, ‘some of the time’ and ‘none of the time’. However, for purposes of this study, the first two categories were collapsed into one group.

### General health and psychological measures

#### General health

Self-assessed general health was measured with a single item *“Overall, how would you rate your health during the past 4 weeks?”* Response options were ‘1 = Excellent’, ‘2 = Very good’, ‘3 = Good’, ‘4 = Fair’, ‘5 = Poor’, and ‘6 = Very poor’. Further, self-rated disability status was measured with a single question *“Do you have a disability, injury or health condition that has lasted or is likely to last 12 months or more?”* Response options were ‘yes’ and ‘no’. These two general health measures collected at Wave 1 were used for this analysis.

#### Traumatic events

Respondents were presented with a list of nine potentially traumatic events and were asked whether they had experienced or witnessed any of them, based on their own personal experience. Examples of the events listed included extreme living conditions (e.g. lack of food, water, shelter or medicine), kidnapping or imprisonment, murder, unnatural death or disappearance of family or friends, torture, and other events. The category ‘other’ could also be selected. Respondents had the option of selecting ‘does not apply’, ‘I don’t know’ or ‘prefer not to say’. These responses were coded ‘no response’. This question was available in Wave 3.

#### Mental health

Mental health symptoms were measured using two scales that have been extensively applied to cross cultural populations. The Kessler Screening Scale for Psychological Distress (K6) [23, 24] is a measure of psychological distress experienced in the last four weeks. It generates possible scores ranging from 6 to 30 and has been validated against clinical diagnoses. The PTSD-8, which is derived from the Harvard Trauma Questionnaire (HTQ), measures symptoms consistent with PTSD such as intrusion, avoidance and hypervigilance and is based on respondents’ self-report of feelings over the past week. Respondents were classified as likely having PTSD if they answered ‘sometimes’ or ‘most of the time’ to at least one item in each of the domains (i.e. intrusion, avoidance, hypervigilance) [25]. For purposes of this study, data from both scales at Wave 1 were used.

### Dependent variable

#### Professional help-seeking and frequency of help received

Professional help-seeking was measured using a single question *“Since being in Australia, have you received help from a professional, such as a doctor, counsellor or psychologist to help you deal with emotional problems?”*. Response options for this question were ‘yes’ or ‘no’. Those who selected ‘yes’ were then also asked *“Have you received this help in the last 12 months?”* to determine time frame around help received. Finally, the frequency of receiving help was also asked of those who reported having received help in the last 12 months. These questions were available in Wave 3.

### Statistical analyses

Statistical analyses were conducted using Stata [26]. Descriptive analysis included the estimation of means and standard errors for continuous variables and proportions and frequencies for categorical variables. Significant differences in sample characteristics by country of birth and by whether respondents received professional help were assessed using adjusted Wald test for continuous variables and corrected weighted Pearson chi-squared tests for categorical variables. Descriptive analyses were conducted with the ‘svy’ procedure in Stata and used weighted data. The percentages presented in the descriptive tables are weighted, while the frequencies represent the numbers in the sample; cases with missing values are excluded analysis by analysis. Univariate and multivariate within group logistic regressions were conducted to assess the associations between socio-demographic characteristics and general and mental health measures and the probability of receiving help for emotional problems. Logistic regressions controlled for the clustered nature of the data using the cluster option in Stata. Given that the question on help-seeking at Wave 3 refers to receiving help since arrival, independent variables utilised were all measured at Wave 1 (unless otherwise stated) as this represents the closest time point to the time of arrival.

## Results

Table 1 presents the associations between socio-demographic and clinical characteristics by country of origin and across those who receive help versus those who did not.

**Table 1:** Participant socio-demographic characteristics, general health and psychological measures by country of birth and whether received help for emotional problems

	Iraq (*n* = 803) % (*n;* SE)	Afghanistan (*n* = 485) % (*n;* SE)	*p* value	Total (*n* = 1288) ^a^ % (*n;* SE)	Did not receive help (*n* = 790) % (*n;* SE)	Received help (*n* = 478) % (*n;* SE)	*p* value
Socio-demographic characteristics							
Mean age	37.8 (803; 0.55)	33.2 (485; 0.58)	< 0.001	36.1 (1288; 0.41)	34.1 (790; 0.51)	39.6 (478; 0.67)	< 0.001
Gender							
Female	48.6 (390)	44.4 (218)	0.154	47.0 (608)	45.5 (357)	49.3 (240)	0.191
Male	51.4 (413)	55.6 (267)		53.0 (680)	54.5 (433)	50.7 (238)	
Education							
Never attended school	5.4 (44)	36.2 (182)	< 0.001	16.8 (226)	14.8 (122)	19.6 (97)	0.004
9 or less years school	42.3 (335)	47.9 (226)		44.3 (561)	42.5 (331)	47.9 (223)	
10 or more years schooling	31.7 (251)	13.1 (59)		24.8 (310)	27.3 (207)	20.5 (98)	
Post-school education	20.6 (168)	2.8 (14)		14.1 (182)	15.5 (125)	12.0 (56)	
Location			< 0.001				0.328
Major city	99.8 (800)	86.6 (403)		94.9 (1203)	95.3 (741)	94.2 (443)	
Regional Australia	0.2 (3)	13.5 (82)		5.1 (85)	4.7 (49)	5.8 (35)	
Time in Australia			0.004				0.044
Less than 6 months	83.8 (676)	77.2 (376)		81.4 (1052)	80.0 (629)	84.7 (410)	
6 months or more	16.2 (127)	22.8 (109)		18.6 (236)	20.0 (161)	15.3 (68)	
Importance of religion^			0.018				0.027
Very important	74.2 (595)	72.3 (350)		73.5 (945)	71.1 (557)	78.0 (375)	
Important	22.2 (173)	20.4 (97)		21.5 (270)	23.9 (187)	17.3 (79)	
Not very important/ no religion	3.7 (31)	7.3 (32)		5.0 (63)	5.0 (40)	4.8 (21)	
Frequency difficulty travelling to required locations			0.267				0.003
Always/ most of the time	43.8 (339)	45.3 (219)		44.4 (558)	42.3 (325)	46.9 (219)	
Some of the time	30.7 (243)	33.2 (162)		31.6 (405)	30.3 (239)	34.6 (163)	
Never	25.5 (189)	21.5 (101)		24.0 (290)	27.4 (207)	18.5 (80)	
General health and psychological measures							
Self-rated health			< 0.001				< 0.001
Excellent/ very good	22.2165)	49.3 (230)		32.2 (395)	38.2 (291)	22.5 (101)	
Good/fair	56.7 (455)	41.7 (207)		52.1 (662)	51.6 (411)	49.2 (236)	
Poor/ very poor	21.2 (183)	9.0 (48)		16.7 (231)	10.2 (88)	28.3 (141)	
Disability			< 0.001				< 0.001
Has disability	32.2 (271)	20.5 (105)		27.9 (381)	18.5 (157)	43.8 (216)	
No disability	67.9 (520)	79.5 (373)		72.2 (893)	81.5 (626)	56.2 (255)	
Number of potentially traumatic events			< 0.001				0.004
No response	10.0 (74)	24.2 (112)		15.3 (186)	16.3 (124)	12.3 (55)	
1 event	23.3 (180)	35.9 (171)		28.0 (351)	30.1 (232)	24.4 (113)	
2–3 events	33.1 (271)	23.2 (115)		29.5 (386)	29.0 (232)	30.5 (149)	
4 or more events	33.6 (278)	16.6 (87)		27.3 (365)	24.6 (202)	32.9 (161)	
Probable PTSD			< 0.001				< 0.001
Unlikely PTSD	54.5 (414)	77.7 (358)		62.9 (772)	70.6 (540)	49.6 (221)	
Likely PTSD	45.6 (368)	22.3 (104)		37.1 (472)	29.4 (225)	50.4 (239)	
Psychological distress (K6)			< 0.001				< 0.001
K6 mean score	14.2 (776; 0.22)	11.8 (475; 0.26)		13.3 (1251; 0.17)	12.0 (767; 0.31)	15.5 (464; 0.20)	

BNLA Wave 1 and Wave 3. Weighed percentages, sample n. SE: standard error. Corrected weighted Pearson chi-squared (percentages) and adjusted Wald (means) tests for significant differences by country of birth and by whether received help: *** *p* > 0.001, ** *p* > 0.01, * *p* > 0.05. Variables measured at Wave 1, except for potentially traumatic events and help-seeking. ^ 48.3% (*n* = 602) of the sample were Christians (76.3% (*n* = 597) among Iraqis and 0.9% (5) among Afghans) and 44.8% (*n* = 588) of the sample were Muslims (12.6% (*n* = 109) of Iraqis and 99.1% (479) of Afghans). Among Iraqis 11.0% (*n* = 89) had other religions (including 1 case no religion)

^a^Excludes: 20 participants who did not report whether they had received help; 34 participants who did not report PTSD; 37 participants who did not report K6; 9 participants who did not report education; 14 participants who did not report disability; 10 participants who did not report on the importance of religion; 35 participants who did not report on the frequency of travelling difficulties

The Afghan subgroup was significantly younger (F(1, 1287) = 34.05, *p* < 0.001) and had fewer years of education (F(3.00, 3829.45) = 89.56, *p* < 0.001). Compared with the Iraqi subgroup, a higher percentage of the Afghan sub-group was located in regional towns (F(1, 1287) = 196.32, *p* < 0.001), had been in Australia for 6 months or more at Wave 1 (F(1, 1287) = 8.24, *p* < 0.001) and reported religion as being not important to them (F(2.00, 2553.48) = 4.02, *p* < 0.05) than the Iraqi subgroup. Further, comparatively to those from Iraq, a higher percentage of those from Afghanistan rated their health as excellent to good (F(2.00, 2567.66) = 53.44, *p* < 0.001) and subsequently reported less rated disability (F(1, 1273) = 20.29, p < 0.001). In terms of trauma and psychological measures, the Afghan group had been exposed to fewer potentially traumatic events (F(2.99, 3854.45) = 31.80, p < 0.001), had lower levels of psychological distress (F(1, 1250) = 48.43, p < 0.001) and a lower percentage had probable PTSD (F(1, 1243) = 65.57, p < 0.001) compared to the Iraqi sample. It should be noted however that approximately a quarter of the Afghan group (24.2%) did not respond to the question on exposure to potentially traumatic events. Table 1 also reports on the frequency and mean data for each variable comparing those who received help for emotional problems versus those who did not, however examination of significant differences is reported following Table 3.

Table 2 presents the percentage of help received by county of origin and frequency of help received.

**Table 2:** Percentage of respondents who received help by country of birth and frequency of help received

Variables outcomes	Total (*n* = 1268)		Iraq (*n* = 791)		Afghanistan (*n* = 477)	
Help Received	n	% [CI]	n	% [CI]	n	% [CI]
Received help since arrival	478	36.4 [33.8, 39.1]	297	35.9 [32.6, 39.4]	181	37.2 [32.9, 41.7]
Did not receive help	790	63.6 [60.9, 66.2]	494	64.1 [60.6, 67.4]	296	62.8 [58.3,67.1]
Among those who received help since arrival	Total (*n* = 474)^a^		Iraq (*n* = 293)		Afghanistan (*n* = 181)	
	n	% [CI]	n	% [CI]	n	% [CI]
Received help in the last 12 months	400	83.8 [80.0, 87.0]	251	84.9 [80.0, 88.8]	149	81.9 [75.4, 87.0]
Didn’t receive help in last 12 months	74	16.21 [13.0, 20.0]	42	15.1 [11.2, 20.0]	32	18.1 [13.0, 24.6]
Among those who received help in the last 12 months	Total (*n* = 400)		Iraq (*n* = 251)		Afghanistan (*n* = 149)	
Frequency received help last 12 months	n	% [CI]	n	% [CI]	n	% [CI]
1 or 2 times	105	28.5 [24.1, 33.4]	58	24.6 [19.5, 30.6]	47	35.3 [27.6, 43.8]
3–5 times	89	22.1 [18.3, 26.6]	56	22.2 [17.4, 27.8]	33	22.1 [16.0, 29.7]
6–9 times	77	19.7 [16.0, 24.0]	49	19.8 [15.3, 25.4]	28	19.5 [13.7, 27.0]
10 or more times	117	29.6 [25.2, 34.4]	83	33.4 [27.7, 39.6]	34	23.1 [16.8, 30.9]

BNLA, Wave 3; weighted percentages; sample n. CI: confidence interval. Excludes 20 participants who did not report whether they received help. ^a^ Excludes 4 participants from Iraq who responded ‘prefer not to say’ (n = 2), ‘don’t know’ (n = 1) or ‘does not apply’ (n = 1) in this question

Over a third of respondents reported having received help for emotional problems since arrival to Australia and the numbers were consistent between the two groups (35.9% Iraqi respondents versus 37.2% of Afghan respondents). Of those that reported receiving help, most help was received in last 12 months leading up to the Wave 3 time point, around two-and-a-half years after arrival for most respondents. This percentage did not differ by country of birth. When examining the frequency of help received by respondents, those from Afghanistan were more likely to report receiving help only 1 to 2 times in last 12 months whereas those from Iraq appeared to be more likely to have received help 10 times or more in last 12 months comparatively, nonetheless this difference was not statistically significant (F(3.00, 1159.98) = 2.19, *p* = 0.087).

Table 3 reports the bivariate and multivariate odds ratios and 95% confidence intervals for receiving help for emotional problems according to socio-demographic and clinical characteristics by country of origin. The odds ratios for total sample are presented for reference.

**Table 3:** Odds ratios (95%) for receiving help (vs not receiving help) for emotional problems

Groups	Iraq group (n = 791)				Afghan group (n = 477)				Total group (n = 1268) ^a^			
	Bivariable odds ratios (95% CI)	*p* value	Multivariable odds ratios (95% CI)	*p* value	Bivariable odds ratios (95% CI)	*p* value	Multivariable odds ratios (95% CI)	*p* value	Bivariable odds ratios (95% CI)	*p* value	Multivariable odds ratios (95% CI)	*p* value
Socio-demographic characteristics												
Country of birth												
Iraq (ref.)	–		–		–		–		1.00		1.00	
Afghanistan	–		–		–		–		0.93 (0.0.73–1.22)	0.654	1.38 (0.94–2.03)	0.105
Mean age	1.03 (1.02–1.04)	< 0.001	1.01 (1.00–1.02)	0.186	1.03 (1.02–1.05)	< 0.001	1.02 (1.00–1.04)	0.022	1.03 (1.02–1.04)	< 0.001	1.01 (1.11–1.02)	0.028
Gender												
Male (ref.)	1.00		1.00		1.00		1.00		1.00		1.00	
Female	1.08 (0.83–1.41)	0.582	0.86 (0.61–1.21)	0.381	1.55 (1.08–2.24)	0.019	1.21 (0.76–1.92)	0.429	1.24 (1.00–1.53)	0.055	0.97 (0.74–1.26)	0.804
Education												
Never attended school (ref.)	1.00		1.00		1.00		1.00		1.00		1.00	
9 or less years school	0.77 (0.39–1.51)	0.456	1.64 (0.73–3.66)	0.231	0.68 (0.44–1.03)	0.066	1.01 (0.61–1.65)	0.981	0.86 (0.61–1.19)	0.354	1.13 (0.74–1.71)	0.571
10 or more years schooling	0.45 (0.22–0.92)	0.029	0.76 (0.33–1.76)	0.518	0.73 (0.39–1.35)	0.315	1.03 (0.49–2.16)	0.931	0.62 (0.42–0.90)	0.013	0.69 (0.43–1.13)	0.138
Post-school education	0.42 (0.20–0.86)	0.019	0.56 (0.23–1.34)	0.192	0.77 (0.25–2.39)	0.647	1.02 (0.29–3.62)	0.979	0.57 (0.37–0.89)	0.013	0.52 (0.30–0.91)	0.022
Location												
Major city (ref.)	1.00				1.00		1.00		1.00		1.00	
Regional Australia	0.81 (0.05–13.08)	0.883	2.37 (0.15–37.83)	0.541	1.26 (0.74–2.16)	0.394	0.90 (0.48–1.68)	0.734	1.17 (0.52–1.92)	0.545	1.06 (0.55–2.02)	0.870
Time in Australia												
Less than 6 months (ref.)	1.00		1.00		1.00		1.00		1.00		1.00	
6 months or more	0.76 (0.48–1.20)	0.238	1.19 (0.68–2.08)	0.537	0.54 (0.33–0.89)	0.015	0.74 (0.42–1.31)	0.299	0.66 (0.47–0.92)	0.014	0.95 (0.65–1.38)	0.776
Importance of religion												
Very important (ref.)	1.00		1.00		1.00		1.00		1.00		1.00	
Important	0.64 (0.44–0.93)	0.019	0.84 (0.53–1.33)	0.448	0.52 (0.31–0.89)	0.017	0.60 (0.34–1.05)	0.073	0.59 (0.44–0.81)	0.001	0.69 (0.49–0.96)	0.030
Not very important/ no religion^	0.73 (0.31–1.71)	0.473	1.11 (0.36–3.45)	0.861	0.79 (0.36–1.75)	0.568	0.92 (0.40–2.13)	0.850	0.76 (0.43–1.36)	0.451	0.98 (0.67–1.43)	0.981
Frequency of difficulty travelling to where needs to go												
Never (ref.)	1.00		1.00		1.00		1.00		1.00		1.00	
Some of the time	2.57 (1.65–3.99)	< 0.001	2.19 (1.33–3.60)	0.002	0.98 (0.55–1.75)	0.941	0.71 (0.36–1.41)	0.328	1.79 (1.23–2.54)	0.001	1.29 (0.86–1.91)	0.221
Always/ most of the time	2.19 (1.44–3.32)	< 0.001	1.41 (0.88–2.26)	0.153	1.29 (0.73–2.26)	0.381	0.68 (0.36–1.26)	0.221	1.80 (1.23–2.52)	0.001	1.02 (0.70–1.45)	0.911
General health and psychological measures												
Self-rated health												
Excellent/ very good (ref.)	1.00		1.00		1.00		1.00		1.00		1.00	
Good/ fair	2.15 (1.38–3.34)	0.001	0.89 (0.51–1.54)	0.675	1.82 (1.20–2.77)	0.005	1.31 (0.79–2.17)	0.288	1.75 (1.31–2.35)	< 0.001	1.07 (0.75–1.54)	0.701
Poor/ very poor	7.32 (4.45–12.04)	< 0.001	1.26 (0.62–2.55)	0.518	3.50 (1.70–7.22)	0.001	1.31 (0.52–3.31)	0.563	5.13 (3.54–7.43)	< 0.001	1.52 (0.90–2.56)	0.114
Disability or long-term health condition												
Has disability or health condition (ref.)	1.00		1.00		1.00		1.00		1.00		1.00	
No disability or health condition	0.24 (0.17–0.32)	< 0.001	0.44 (0.28–0.69)	< 0.001	0.42 (0.26–0.68)	< 0.001	0.71 (0.39–1.29)	0.259	0.29 (0.23–0.38)	< 0.001	0.55 (0.39–0.77)	0.001
Number of potentially traumatic events												
1 event (ref.)	1.00		1.00		1.00		1.00		1.00		1.00	
2–3 events	2.06 (1.30–3.25)	0.002	1.92 (1.11–3.31)	0.019	0.64 (0.37–1.11)	0.111	0.57 (0.32–1.03)	0.065	1.29 (0.92–1.83)	0.144	1.13 (0.77–1.67)	0.529
4 or more events	2.15 (1.40–3.31)	0.001	1.67 (0.98–2.84)	0.057	1.39 (0.79–2.42)	0.253	1.15 (0.62–2.14)	0.656	1.65 (1.19–2.29)	0.003	1.29 (0.88–1.90)	0.192
No response	0.98 (0.51–1.88)	0.942	1.28 (0.55–2.97)	0.568	0.67 (0.38–1.18)	0.170	0.71 (0.38–1.31)	0.273	0.83 (0.54–1.27)	0.384	0.90 (0.55–1.46)	0.668
Probable PTSD												
Unlikely PTSD (ref.)	1.00		1.00		1.00		1.00		1.00		1.00	
Likely PTSD	3.43 (2.53–4.64)	< 0.001	1.58 (1.02–2.44)	< 0.039	1.79 (1.11–2.88)	0.017	1.16 (0.67–2.00)	0.603	2.64 (2.07–3.37)	< 0.001	1.42 (1.03–1.96)	0.034
Psychological distress (K6)												
K6 mean score	1.14 (1.10–1.17)	< 0.001	1.09 (1.05–1.13)	< 0.001	1.07 (1.04–1.11)	< 0.001	1.04 (1.00–1.09)	0.058	1.11 (1.08–1.13)	< 0.001	1.07 (1.04–1.10)	< 0.001

BNLA, Wave 1 and Wave 3. Potentially traumatic events and help-seeking measured at Wave 3. All other independent variables measured at Wave 1

^a^ Excludes: 34 participants who did not report PTSD; 37 participants who did not report K6; 9 participants who did not report education; 14 participants who did not report disability; 10 participants who did not report on the importance of religion; 35 participants who did not report on the frequency of travelling difficulties. ^Includes one respondent with no religion

### Iraqi subgroup

A number of significant univariate associations were found for the Iraqi subgroup. Firstly, higher mean age and greater difficulty with transportation were positively associated with help received, however higher levels of education and those who considered religion as important (versus those who thought it was very important) were less likely to receive help. Finally, as expected, those with poorer self-rated health, exposure to more traumatic events, probable PTSD and higher rates of psychological distress were all more likely to have received help. Not surprisingly, those with no disability were less likely to have received help. When a multivariable model was examined fewer associations remained. Notably, those who had difficulty with transport, had been exposed to at least 2 to 3 traumatic events, had probable PTSD and higher rates of psychological distress were more likely to have received help, whereas those reporting no disability were less likely to have received help.

### Afghan subgroup

A similar pattern to that found for the Iraqi subgroup was noted for the Afghan subgroup in the univariate analysis. Namely, age, poorer self-rated health, probable PTSD and higher distress levels were positively associated with receiving help for emotional problems, while those with no reported disability were less likely to have received help. In addition, women were more likely to have received help then men in the Afghan group whereas length of residence in Australia was negatively associated with help received in this subgroup. Interestingly, when these variables were included in the multivariable logistic regression model only age remained positively associated with help received (psychological distress was marginally significant *p* = 0.058).

## Discussion

This paper sought to investigate professional help received in two refugee populations, and determine what factors are associated with help received for emotional problems in one of the largest longitudinal datasets of its kind. The importance of understanding factors associated with professional help-seeking behaviours for mental health problems has been previously highlighted as a possible means of developing programs to reduce the associated public burden of mental health disorders [16]. This is particularly important among refugees, given the high prevalence of mental disorders among this population, originating from pre-migration traumatic experiences and the stressors of settlement [5]. Further, research on the prevalence of mental illness and access to professional help-seeking amongst general community in Australia [27] has demonstrated that despite a high prevalence of mental illness, most people do not access professional health care. Similar patterns have been found for those from refugee backgrounds [10, 11].

Overall, this study’s findings were partly consistent with our previous studies demonstrating an association between measures of mental health status, namely, general psychological distress, PTSD symptoms, functional disability and help-seeking [11–13]. Specifically, in the Iraqi multivariate analysis it was noted that exposure to traumatic events, probable PTSD and higher levels of psychological distress were associated with increased help-seeking. Further, those Iraqis with lower levels of functional disability had lower levels of help-seeking. However, when considering these factors in the Afghan subgroup there were no such associations between mental health status, including functional disability, and help-seeking found at the multivariate level. These findings have several implications. Firstly, we have previously argued for the need for mental health promotion and early intervention programs for resettled refugees to be tailored to the characteristics of the population concerned [28] and once again this is supported by the findings of this paper. Clearly, the influence of differing cultural and religious backgrounds on how refugee communities view and respond to mental health problems should not be underestimated. Secondly, as there is now good evidence from community-based research that early, appropriate help-seeking is associated with improved mental health outcomes [29] the finding of higher help-seeking amongst the Iraqi subgroup who were symptomatic or had signs of distress or disability, while in itself intuitively correct does not discount the need to promote early help-seeking amongst those who need it. As such efforts to develop tailored mental health promotion programs that promote an understanding that early intervention can lead to better outcomes should be a priority.

When socio-demographic factors were taken into account in the multivariate models, age was found to be positively associated with help-seeking in the Afghan subgroup, whereas higher levels of education was found to be negatively associated with help-seeking in the Iraqi group, albeit at bivariate level only. Although we have not reported such associations in our previous studies, in a recent systematic review [16] both age and levels of education were consistently reported to be factors influencing help-seeking for depression. However, it should be noted that higher levels of education were noted to be associated with more likelihood of help-seeking in the systematic review [16] implying that the refugee groups may be differing from the general pattern observed in this regard. Our findings suggest that additional attention should be paid to targeting the younger refugee populations and those who are more highly educated when mental health promotion programs are developed. The finding that those who reported having travel difficulty were more likely to seek help within the Iraqi group was not in the direction initially expected. Having transport difficulties can be a barrier for accessing different types of services, including health services, and it is well known that most refugees, including the BNLA sample, commonly resettle in areas of lower socio-economic status that is typically limited in public transport options. Thus our finding is difficult to interpret and may warrant further re-examination in subsequent survey waves. The bivariate association between help-seeking and religion, where it was noted those who considered religion as *important* were less likely to seek help than those who reported religion as *very important* also warrants further consideration. One postulated explanation may be that those who have higher levels of religiosity are more connected to their communities through religious organisations allowing for knowledge exchange about resources and services that are available thus facilitating help-seeking. Further, we have previously reported on the existence of the duality of treatment beliefs when examining mental health literacy in Iraqi [30] and Afghan [28] populations and this association supplements this conclusion. As such the engagement of religious and spiritual leaders in mental health promotion activities should be a matter of priority as they often act as gatekeepers in refugee communities.

Our finding that half of our total sample (50.4%) with probable PTSD reported seeking help for emotional problems is encouraging and appears higher than that reported in the 2007 National Survey of Mental Health and Wellbeing (NSMHWB), where it was noted that only one-third (34.9%) of people with a mental disorder used health services for mental health problems in the 12 months prior to the interview [27]. Nonetheless it still is far from ideal, where we would argue that most if not all those who are demonstrating symptoms of probable PTSD would benefit from seeking help. Frequency of help received is another interesting finding from this study. A recent meta-analysis on treatment for PTSD in refugees indicated that effect sizes for symptom reduction doubled in those who received 20 sessions or more [31]. This would seem to suggest that most of our refugees are not engaging with treatment at the rate that would result in best treatment outcomes and again presents an opportunity and challenge (given the additional funding needed for more intense treatment) to both policy makers and clinicians who are working with these groups.

Several limitations of the study should be noted. Firstly, the survey utilised self-report measures of both mental health status and these can be the subject of general bias such as recall and social desirability, in addition to more specific issues associated with translational and transcultural bias which may have also affected responses. Nonetheless the K6 and PTSD-8 (derived from the HTQ) have been extensively utilised in cross cultural settings and thus offers some reassurance in this regard. Further, the use of single items to assess measures such as physical health, disability, self-sufficiency and help-seeking are not ideal but stem from pragmatic reasons to ensure a survey that is able to be completed in an acceptable timeframe. Additional information on actual help received sourced from health records would provide further insights. Strengths of this study is that it is the first large and broadly representative study on two of the largest refugee groups that have been resettled in Australia in the last decade. As such the findings are likely to be robust and provide a clear window for targeted mental health promotion programs for these populations.

## Conclusions

This is first study to report on help-seeking for emotional problems in two large samples of Iraqi and Afghan refugees in Australia. Our findings indicate that while half of those with probable PTSD are reporting to be seeking help, the level of engagement as measured by frequency of help received remains far from ideal according to research on psychological interventions for refugees. Clear associations between both cultural and structural factors and help-seeking were found. Consequently, our findings provide direction on areas where targeted mental health promotion programs are required such as those who are younger or have higher levels of education. Policy makers and clinicians should note the importance of such factors when working with refugees in order to optimise their resettlement outcomes.

## Data Availability

The BNLA Release 3.0 dataset used in this study is publicly available and accessible by authorised researchers who have obtained permission from the Department of Social Services.

## Abbreviations

AIFS: Australian Institute of Family Studies
BNLA: Building a New Life in Australia
DSS: Department of Social Services
HTQ: Harvard trauma questionnaire
K6: Kessler Psychological Distress Scale - 6
MDE: Major depressive episode
NSMHWB: Australian National Survey of Mental Health and Wellbeing
PTSD: Posttraumatic stress disorder
UNHCR: United Nations High Commissioner for Refugees
